# Occupational exposure of goat farm workers to particulate matter and endotoxin

**DOI:** 10.1093/annweh/wxag020

**Published:** 2026-04-06

**Authors:** Aniek Lotterman, Ifeoluwa Olufotebi, Inge M Wouters, Albert Winkel, Lidwien A M Smit, Myrna M T de Rooij

**Affiliations:** Institute for Risk Assessment Sciences, Utrecht University, PO Box 80178, 3508 TD Utrecht, The Netherlands; Institute for Risk Assessment Sciences, Utrecht University, PO Box 80178, 3508 TD Utrecht, The Netherlands; Institute for Risk Assessment Sciences, Utrecht University, PO Box 80178, 3508 TD Utrecht, The Netherlands; Wageningen Livestock Research, Wageningen University & Research, PO Box 338, 6700 AH Wageningen, The Netherlands; Institute for Risk Assessment Sciences, Utrecht University, PO Box 80178, 3508 TD Utrecht, The Netherlands; Institute for Risk Assessment Sciences, Utrecht University, PO Box 80178, 3508 TD Utrecht, The Netherlands

**Keywords:** intensive livestock farming, bioaerosols, respiratory health, occupational health, exposure assessment

## Abstract

**Introduction:**

Intensive livestock farming exposes workers to elevated levels of particulate matter (PM) and endotoxins, increasing respiratory health risks. Although personal exposure has occasionally been investigated in the 3 major livestock sectors—cattle, pigs, and poultry—emerging sectors such as intensive goat farming remain understudied. This study aimed to quantify personal exposure to inhalable dust, PM_10_, and endotoxins among workers on Dutch dairy goat farms and to explore associations between exposure levels, specific work tasks, and farm characteristics.

**Methods:**

Repeated personal air sampling was conducted among 41 participants working at 15 goat farms. Inhalable dust and PM_10_ samples were collected using filter-based methods attached to portable air pumps. For all inhalable dust and PM_10_ samples, PM mass concentrations were determined by gravimetrical analyses and endotoxin concentrations with the Limulus-Amebocyte-Lysate assay. Determinants of exposure levels were analyzed by linear mixed modeling.

**Results:**

Inhalable dust concentrations showed a median of 0.966 mg/m^3^ (range: 0.228 to 3.093), with a median endotoxin concentration of 612 EU/m^3^ (range: 48 to 7,818). For the PM_10_ concentrations, a median of 0.376 mg/m^3^ (range: 0.070 to 1.233) was observed, with a median endotoxin concentration of 700 EU/m^3^ (range: 8 to 2,886). In total, 90% of the samples exceeded recommended occupational exposure limits for endotoxin (>90 EU/m^3^). PM_10_ and inhalable dust concentrations were strongly correlated (Pearson *r* = 0.71), as were endotoxin concentrations in both fractions (Pearson *r* = 0.71). Exposure to PM and endotoxin varied significantly between farms, within farms and within workers. Overall, highest exposures were recorded for workers with milking as a primary job task.

**Discussion and conclusions:**

Goat farm workers are exposed to substantial levels of PM and endotoxins during routine work activities. On average, concentrations exceeded those reported for dairy cattle farm workers, yet remained lower than levels typically observed in pig and poultry farming. The observed considerable variation in exposure both between farms and among individual workers necessitates future research and more detailed microbiological characterization of air samples on determinants of exposure, to guide appropriate measures in husbandry and practices at goat farms. This research highlights that in emerging intensive livestock farming sectors, increased exposure to PM and endotoxins—and related health effects—can be expected among workers unless reduction of these exposures is explicitly addressed in the development of agricultural policies and practices.

What's Important About This Paper? This is the first study to perform personal exposure monitoring among goat farmers, and found that goat farmers experience elevated exposures to particulate matter and endotoxins. This study clearly shows that goat farmers belong to a highly exposed occupational group, and provides guidance for future research to deeper investigate the influence of farm characteristics, management, and work activities on farmers’ exposures.

## Introduction

Air pollution levels can be high inside intensive livestock houses and are characterized by high concentrations of particulate matter of biological origin (Spaan et al. 2006). These so-called bioaerosols, small airborne particles and droplets of biological origin, originate from highly abundant organic matter in livestock houses, including animals’ manure, urine, feathers, skin, and hair debris, besides bedding material and feed (Aarnink et al. 2011b; Cambra-López et al. 2011a). As a consequence, livestock farmers are at increased risk of respiratory diseases due to their occupational exposure to particulate matter (Portengen et al. 2005; Liebers et al. 2006, 2008; Eduard et al. 2009; American Thoracic Society 2012; Guillien et al. 2019; Sigsgaard et al. 2020). Personal exposure assessment studies, which are essential to better characterize and understand occupational health risks, have primarily been performed for the major types of livestock farming being pig, poultry, and cattle farming. With the rise of other types of intensive livestock farming, such as goats and poultry beyond chicken (eg geese and ducks) (Ismoyowati et al. 2019; FAOSTAT), research on this needs to be expanded.

Growth and intensification of the goat farming sector is an ongoing trend that is expected to develop worldwide. Not only because popularity of goat dairy products has been increasing, but especially given that goat farming appears better adapted to future challenges related to climate change (FAOSTAT). Goats are more resilient to high temperatures than cattle, and have a smaller carbon footprint (FAO, Global Livestock Environmental Assessment Model 2017; Pragna et al. 2018; Nazan Koluman 2023). The Netherlands is a major livestock-producing country globally and a leader in agricultural developments. Intensification of goat farming already emerged here in 2000, with the total number of dairy goats increasing from roughly 100,000 (on 838 farms) in 2000 to almost 500,000 (on 651 farms) in 2023 (WSER 2025). Intensive goat farms generally use deep-litter housing (bedded pack barn), where fresh straw is regularly added on top to maintain a dry surface for the animals. Most houses rely on natural ventilation, with partially open side walls covered with bird netting. And air is ventilated outwards through an open roof ridge. However, the implementation of mechanical ventilation systems is becoming more common. Findings from emission research performed at goat farms showed that concentrations of particulate matter (PM) can be high inside the farm (mean PM > 0.28 mg/m^3^) (Aarnink et al. 2011a, 2014). These studies also showed that endotoxins—potent microbiological toxins—were highly abundant in the PM and that endotoxin content differed between size fractions. It is well known that for more dynamic jobs, like farming, stationary air sampling typically underestimates personal exposure (Wallace 2000; Basinas et al. 2015). Consequently, comprehensive personal air sampling, including different size fractions, is essential to assess occupational exposure in goat farmers.

Occupational exposure studies performed in other intensive farming systems indicated PM and endotoxins as key exposures of interest. Endotoxins, also known as lipopolysaccharides, are components of the cell wall of Gram-negative bacteria and are potent inducers of a pro-inflammatory immune response (Poole and Romberger 2012). High personal inhalable dust concentrations were reported, with on average 4 mg/m^3^ for poultry farm workers, 3.4 mg/m^3^ for pig farm workers, and 1 mg/m^3^ for dairy cattle farm workers (Eduard et al. 2004; Samadi et al. 2009; Bønløkke et al. 2012; Basinas et al. 2013, 2014; Pfister et al. 2018). High personal exposures to endotoxin were also observed, often exceeding the Dutch Expert Committee on Occupational Safety (DECOS) proposed health-based occupational exposure limit for endotoxin exposure (of 90 endotoxin units [EU]/m^3^) by up to 90-fold (DECOS 1998). Reviews on farmers’ exposure studies report frequent exceedances of this limit, with endotoxin concentrations in free-ranging hen houses reported at an average of 2,140 EU/m^3^ (Spaan et al. 2006; Farokhi et al. 2018; Liebers et al. 2020). Thus, goat farmers are expected to experience elevated personal exposure to PM and endotoxins, given the husbandry system and labor-intensive farming practices (Cambra-López et al. 2010, 2011a).

The anticipated expansion of intensive goat farming will likely result in a larger goat farmer population and thus more people being occupationally exposed to elevated levels of PM and endotoxins. Therefore, it is essential to study personal exposure to enhance characterization of occupational health risks, which will enable better risk management to ultimately promote the health of this workforce. In our study, first insights into the personal exposure of goat farm workers to PM and endotoxins were obtained. We performed comprehensive personal air sampling to measure and compare concentrations of inhalable dust, and PM_10_ (particulate matter smaller than 10 μm), and, for both size fractions, concentrations of endotoxins. In addition to inhalable dust, a standard size fraction in occupational exposure research, we also included PM_10_ as these particles can penetrate into the deeper airways and are therefore primarily associated with lower respiratory tract effects. Furthermore, we explored relationships between exposures, work profiles, and farm characteristics.

## Methods

This study was part of the Livestock Farming and Neighboring Residents’ Health research program (Van der Giessen et al. 2024), which has investigated health effects associated with residing in close proximity to livestock farms, since 2013. In 2018, a questionnaire was sent to all registered goat farms (*n* = 350) in the Netherlands to gather insights into farm and management practices (Van der Giessen et al. 2024). For the current study, invites were sent to all participants (*n* = 87) of this questionnaire survey which resulted in 11 participating goat farms. Recruitment through networking resulted in participation of 4 additional goat farms (who did not participate in the 2018 questionnaire survey). To be eligible for inclusion, each farm was required to have at least 2 individuals working on-site who were willing to participate. Each farm was visited twice, with at least 1 wk between visits, during the period from June until December 2021.

**Figure 1:**
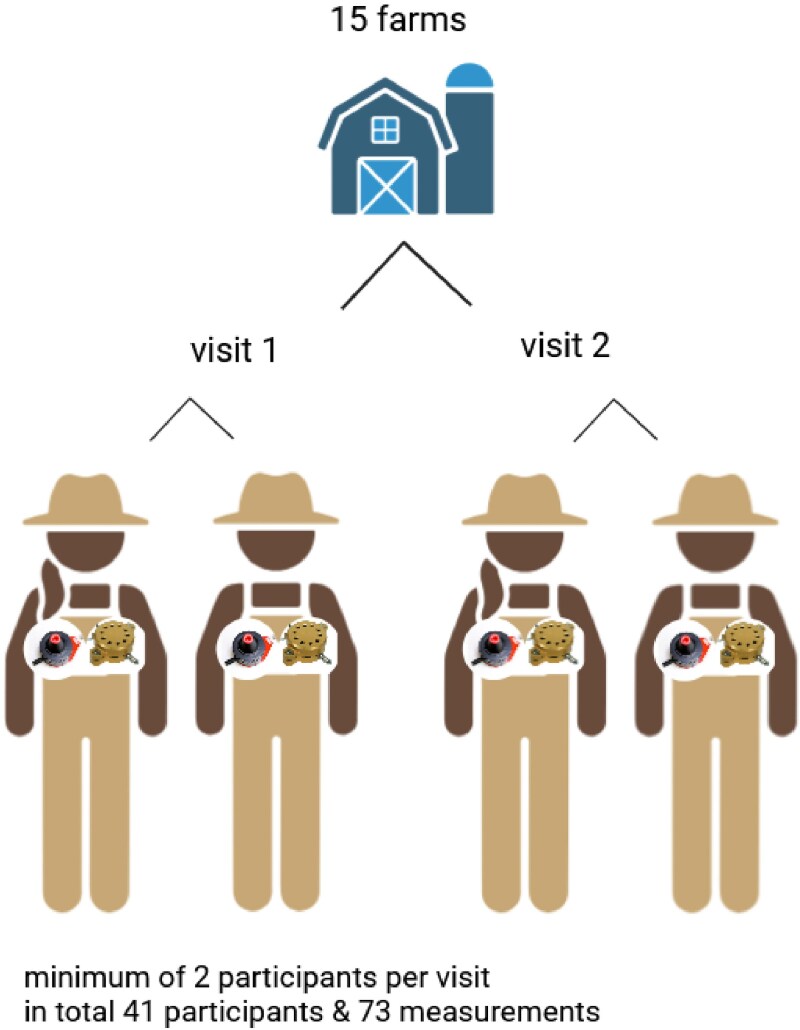
Graphical representation of the study design. Created with BioRender.com.

During each visit, all farmers and farm workers who consented to participate were included in the measurements (Fig. 1). Given the consistency of the daily workforce, most, but not all, workers were measured twice. Visits were scheduled on regular working days (Monday–Friday) and were avoided during infrequent activities such as assisting veterinarians or (re-)penning animals. Measurements covered the entire working day for each participant.

Participants recorded their work activities in a task log (Table S1) during the day of sampling. In addition, the farm manager was asked to complete a questionnaire to provide information on farm characteristics and management practices.

### Measurements

Personal air samples were collected in the breathing zone of the participating farmers/farm workers. All participants were equipped with a waistbelt carrying 2 portable air pumps (Gillian GilAir 5 pump, Sensidyne, St. Petersburg, USA), connected through flexible tubes to the sampling heads. One pump was connected to an inhalable dust sampler (CIS; conicle inhalable sampler; JS Holdings, Stevenage, UK; flow rate: 3.5 L/min) collecting PM with a D_50_ nominal aerodynamic diameter of 100 μm, and the other to a PM_10_ sampler (PEM; Personal Environmental Monitor, MSP90 Corporation, Minnesota, USA; flow rate: 4.0 L/min) collecting PM with a D_50_ nominal aerodynamic of 10 μm. Both samplers contained a Teflon filter (Teflon W/ring 37 mm with 2-µm pore size; Pall Corporation, Michigan, Ann Arbor, USA) to capture the particles. Air flow was calibrated at the start of each sampling day, using a rotameter. The flow rate was measured again at the end of the sampling day. Sample heads were transported to the lab at 4 °C. Within 6 h after collection, the filters were stored at −80 °C until further processing. In addition, 1 field blank of each type (inhalable dust and PM_10_) was collected each sampling day for gravimetrical adjustment and as a control for contamination.

### Laboratory analyses

The particle mass was determined gravimetrically using a micro balance (1-µg precision) and filter controls, following the protocol of the Institute of Risk Assessment Sciences (IRAS) of Utrecht University (IRAS 2009). In brief, prior to both pre- and postweighing, filters were conditioned for 24 h in a climate-controlled room (temperature 21 ± 0.5 °C; relative humidity 35 ± 5%). Particle mass concentrations were expressed in milligrams per cubic meter of air (mg/m^3^). The limit of detection (LOD) was defined as 3 times the standard deviation of the mean field blank PM weight, corresponding to 0.032 mg/m^3^ for inhalable dust and 0.119 mg/m^3^ for PM_10_.

Within 24 h after weighing, filters were processed for endotoxin extraction. In the meantime, filters were stored at 4 °C. Before extraction, filters were acclimatized to room temperature (at least 1 h). The filters were placed into 5-mL Eppendorf tubes and extracted by adding 2.5 mL of pyrogen-free water containing 0.05% Tween 20. The tubes were placed in an end-over-end roller for 60 min and centrifuged at 1,000 × *g* for 15 min (Spaan et al. 2008). Subsequently, 0.5 mL of the supernatant was collected per sample and vortexed, aliquoted, and stored at −20 °C for endotoxin analysis. Endotoxin levels were determined using the Limulus-Amebocyte-Lysate assay (LAL assay) as described previously (Spaan et al. 2008). The LAL assay is a biokinetic assay assessing the toxigenicity/pyrogenicity of the endotoxin present, which is important for extrapolation to potential health effects. Briefly, the sample was diluted (inhalable dust samples 2,500 times and PM_10_ samples 500 times), and a quantitative chromogenic LAL assay was performed (Lonza, Walkersville, Maryland, USA; LAL-lysate lot number VL066WHPV6). The outcomes were expressed in endotoxin units (EU) per cubic meter of sampled air (EU/m^3^), all samples were above the LOD. A subset of field blanks was analyzed (46 for both PM fractions combined), and results showed all were below the LOD (42 EU/m^3^ for inhalable dust fraction and 7.5 EU/m^3^ for PM_10_ fraction), indicating no contamination issues.

### Data analysis

Statistical analyses were performed in R Studio (Version 4.3.1) (Posit team 2025). There were 3 different types of ventilation identified for the included farms: (i) only mechanical ventilation in all animal houses, (ii) only natural ventilation in all animal houses, and (iii) a combination of natural and mechanical ventilation which was labeled as “mixed.” Given the common staff management structures and allocation of activities on Dutch dairy goat farms, 3 work profiles can be distinguished based on the part of the work shift spend on the task milking (mainly, some, none). Participants with the work profile “main milking” spent more than half of their shift milking goats, remaining inside animal houses. Those with the work profile “also milking” spent less time on milking and predominantly did other tasks, such as animal care, feeding, or maintenance. Participants with the work profile “no milking” were mainly farm owners or managers; their activities included animal care and various general farm-related tasks outside the animal housing (eg administration and maintenance). At each visit, every participant was classified into 1 of these 3 work profiles for their shift of that day; see Table S2 for proportions of time spent on tasks based on total sampling duration per participant, per sampling moment.

Distributions were carefully examined for each of the 4 exposure outcomes: concentrations of inhalable dust and PM_10_ mass, and concentrations of endotoxins in both inhalable dust and PM_10_ fraction. As all followed approximately a normal distribution, all statistical analyses were conducted without prior log-transformation of the concentration values. For endotoxin concentration in the inhalable dust fraction, we identified 2 extreme outliers. Because the unrealistic values of these 2 samples could not be retested as aliquots were finished, we therefore decided to winsorize these to the value of the highest observation thereafter (97th percentile). Pearson correlation analyses were performed between all 4 exposure outcomes. Analyses of variance to quantify variance components were performed using the lmer function from the packages lme4 and lmerTest (for Satterthwaite's approximated *P*-values for a small sample size; Kuznetsova et al. 2017). To analyze associations with work profile and ventilation system, linear mixed-effects modeling with random intercepts per farm and participant was performed using lmer and lmerTest. Model assumptions were checked and met, and statistical significance was defined as *P* < 0.05 with trends indicated for *P* < 0.1.

## Results

### Descriptives of goat farms and personal exposure

Of the 15 goat farms included, 14 were conventional farms and 1 organic. All but 1 farm practiced continued milking, meaning there is no drying off period for the goats before kidding. Considerable differences existed between farms regarding (i) feed and feeding method, (ii) manure storage method and duration, (iii) type of farm, and (iv) ventilation system (details in Table S3). For example, manure storage methods such as “Covered (air permeable or airtight)” were reported on 8 farms, “Roofed” was reported on 1 farm, “Uncovered/open” was reported on 5 farms, and 4 farms reported manure storage inside a stable or silo.

**Figure 2:**
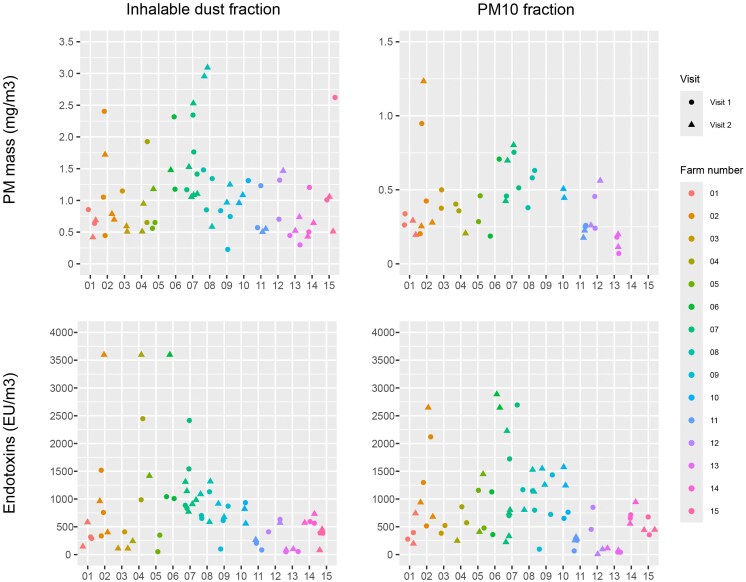
Plot of all exposure concentrations measured by personal air sampling of farm workers during 2 visits at 15 Dutch goat farms colored by farm. Note. For farms 9, 14, and 15, no single PM_10_ mass could be determined due to technical error.

In total, 41 different goat farm workers were included, and samples were taken on 73 typical working days. Thirty-one workers were measured during 2 working days; 10 workers were measured during 1 working day. Sampling was performed for an average of 6 h (range: 1.5 to 9). See Fig. 2 for all exposure concentrations measured. The median inhalable dust mass concentration was 0.966 mg/m^3^ (range 0.228 to 3.093) with a median endotoxin concentration of 612 EU/m^3^ (range 48 to 7,818). The advised occupational exposure limit for endotoxins in inhalable dust was exceeded in 90% (59 out of 65) of the samples. A median PM_10_ mass concentration of 0.376 mg/m^3^ was measured (range 0.070 to 1.233) with a median endotoxin concentration of 700 EU/m^3^ (range 8 to 2,886). The median endotoxin load per mg dust was 582 EU/mg in the inhalable dust fraction (Q1: 382 and Q3: 932) and 1,676 EU/mg in the PM_10_ fraction (Q1: 1,052 and Q3: 2,535); thus, the highest load of endotoxin was found in the PM_10_ fraction.

### Variance and correlation

Variance analyses showed that for all 4 exposure outcomes, most of the variance occurs at the within-worker level, ie variation over time (Table 1), representing between 49% and 63% of the total variance in concentration levels. For inhalable dust, the within-farm level is the next most important variance component, while for endotoxin in PM_10_ fraction, this is the between-farm level. For the other exposure outcomes, the within- and between-farm levels contribute to a similar extent.

**Table 1:** Variance analysis of personal PM (mg/m^3^) and endotoxin (EU/m^3^) exposure.

	Inhalable dust fraction		PM_10_ fraction	
	PM mass (mg/m^3^)	Endotoxin (EU/m^3^)	PM mass (mg/m^3^)	Endotoxin (EU/m^3^)
Between farm (*n* = 15)	12.9%	21.6%	19.5%	26.8%
Within farm (between worker, *n* = 41)	38.2%	23.0%	18.9%	10.0%
Within worker (between visits, *n* = 2)	48.9%	55.4%	61.6%	63.2%

**Figure 3:**
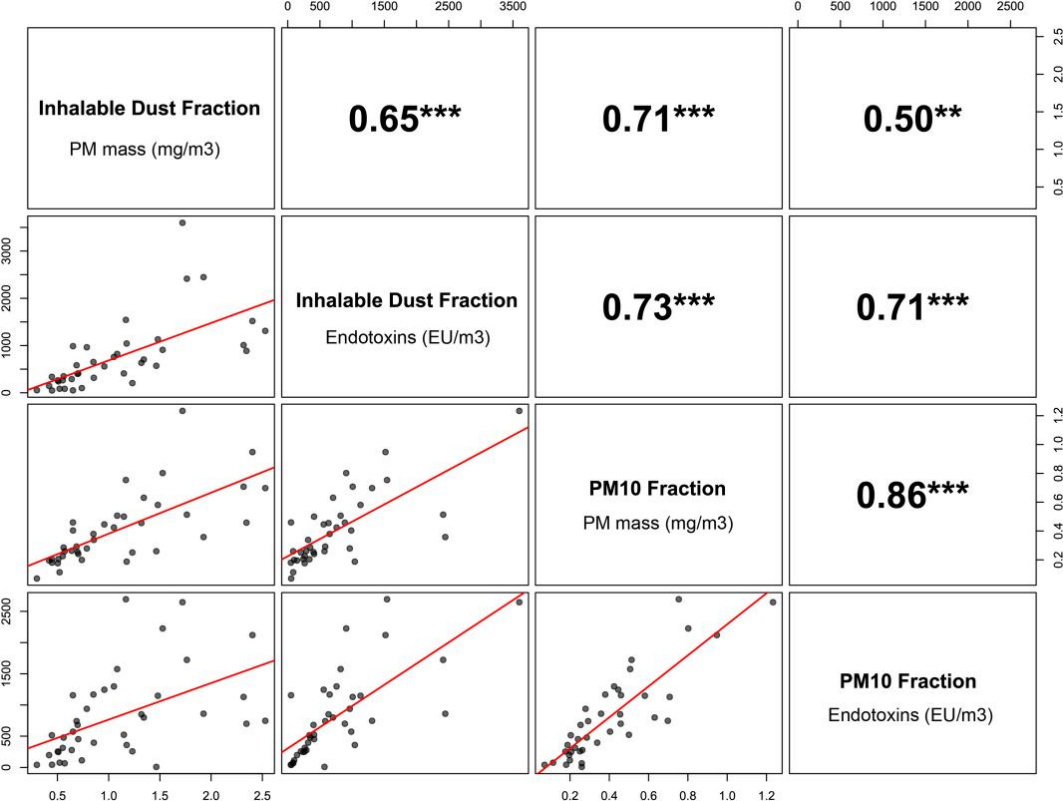
Correlation between all 4 exposure outcomes plotted against each other in the lower panel, together with the Pearson correlation coefficient in the upper panel. Axes are presenting the concentrations for PM in mg/m^3^ and for endotoxin in EU/m^3^. The red line shows a linear curve fitted on the data points. **P-value <0.01. ***P-value <0.001.

The Pearson correlation matrix between all 4 exposure outcomes is shown in Fig. 3. The PM mass concentrations of both size fractions, inhalable dust and PM_10_, were strongly correlated with a Pearson's *r* coefficient of 0.71 (*P* < 0.001). Likewise, the endotoxin concentrations in both size fractions were strongly correlated (*r* = 0.71, *P* < 0.001). PM mass concentration correlated strongly with the endotoxin concentration in the PM_10_ fraction (*r* = 0.86, *P* < 0.001) but only moderately in the inhalable dust fraction (*r* = 0.65, *P* < 0.001).

### Work profiles and ventilation systems

**Figure 4:**
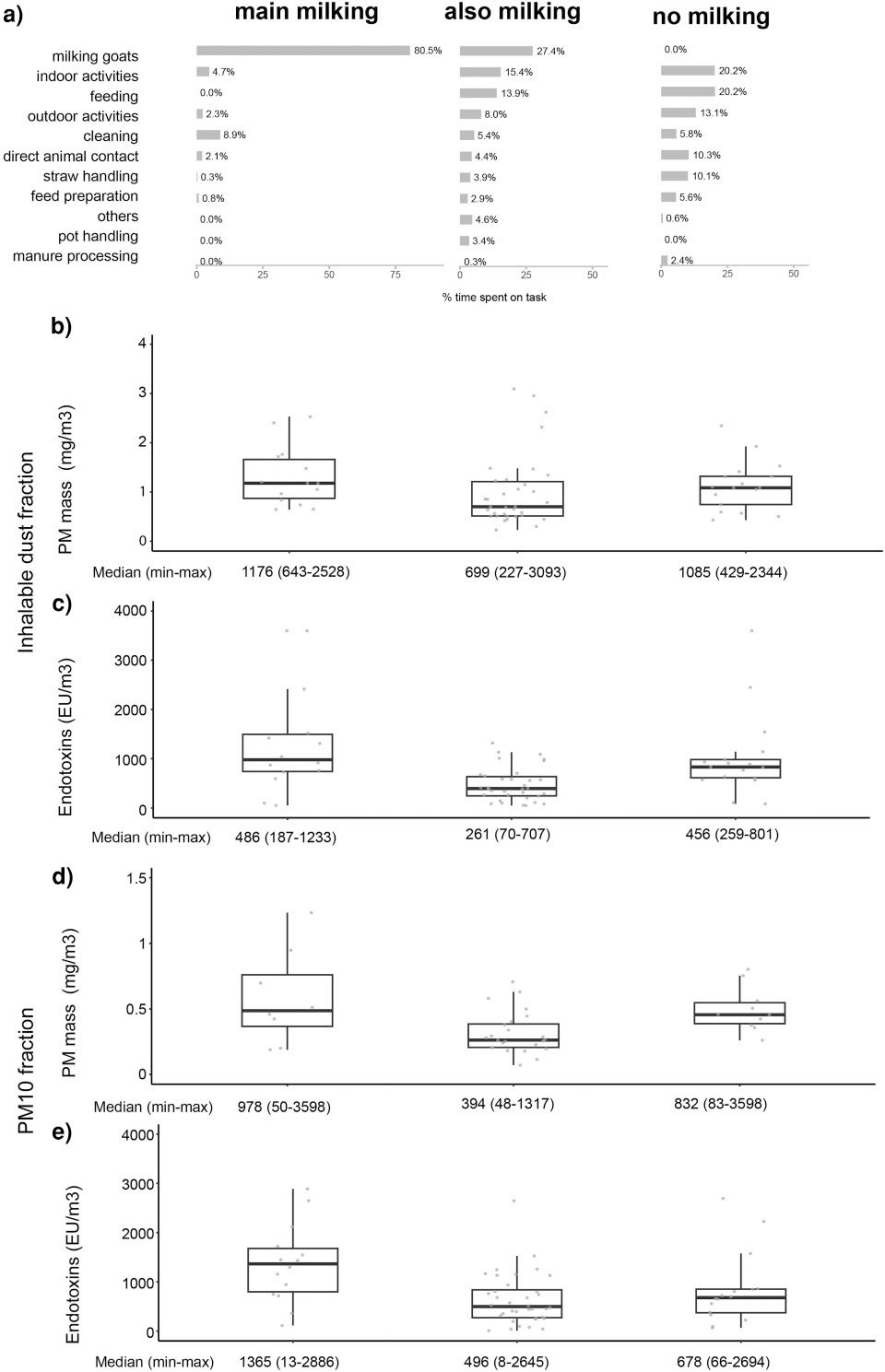
Box plots showing for every exposure the distribution in measured concentrations per work profile (“main milking” on the left, “also milking” in the middle, “no milking” on the right), for the inhalable dust fraction PM mass is shown in panel B and endotoxins in panel C, and for the PM_10_ fraction PM mass is shown in panel D and endotoxins in panel E. Panel A shows bar charts illustrating, for each work profile, the proportion of the work shift spent on different tasks on average.

Work profiles of participants were generally consistent over the farm visits. For the 31 workers with repeated measurements, 27 had the same work profile for both sampling moments (“main milking” [*n* = 6], “also milking” [*n* = 14], and “no milking” [*n* = 7]). Three participants were categorized as “no milking” on 1 workday and “also milking” on the other, while 1 participant was categorized as “main milking” on 1 workday and “also milking” on the other. Figure 3, panel A, presents bar charts showing the average proportion of the work shift spent on various tasks for each work profile. Concentrations of the 4 exposure outcomes varied between work profiles, as shown in Fig. 4 panels B–E, with highest median concentrations observed for the “main milking” profile.

Results of the mixed model showed that PM_10_ mass and endotoxin exposures in workers with the “main milking” profile were significantly higher than in workers with the “also milking.” Compared with the “no milking” profile, a trend toward higher endotoxin concentration in the PM_10_ fraction was observed (*P* < 0.1; Table S4).

On farms with mechanical ventilation systems, higher median and maximum concentrations compared to natural ventilated farms were observed for all 4 exposure outcomes (see Fig. S1). For endotoxins in the PM_10_ fraction, the biggest difference in median concentration was observed, namely 1,148 versus 667 EU/m^3^. Yet, only borderline statistically significant associations between ventilation system and exposure concentrations were identified in the mixed model (*P* = 0.056, est. −618).

## Discussion and conclusions

### Main findings

In this personal exposure assessment study, we found high concentrations of PM and endotoxin during a typical workday of goat farm workers. Overall, between and within farm variance was substantial, and endotoxin content was higher per mg PM_10_ than in inhalable dust. Notable differences across work profiles were observed, with the highest exposures generally occurring in workers whose primary task was milking. Personal endotoxin and PM_10_ concentrations were considerably higher (1.5 to 1.7 times) than those reported in previous studies at goat farms that used stationary air sampling (and/or air sampling at the exhaust) (Winkel et al. 2015). This was in line with expectations and emphasizes the importance of personal air sampling for characterizing occupational exposures. Previous research in other livestock farming populations also demonstrated higher personal exposure levels compared to concentrations measured with stationary sampling inside livestock houses (Dungan 2010; Basinas et al. 2015). This is a common observation in occupational research when tasks are performed close to the source, where additional dust is generated (Wallace 2000), underpinning the value of personal sampling to assess occupational exposure.

We compared our personal exposure measurements in goat farm workers with those in other livestock farmers. These comparisons are, however, only indicative due to the typically small sample sizes in personal exposure studies and the considerable variability observed between farms (Eduard et al. 2004; Samadi et al. 2009; Kirychuk et al. 2010; Bønløkke et al. 2012; Basinas et al. 2013, 2014; Pfister et al. 2018).

Inhalable dust concentrations for goat farm workers were in the same range (1.0 mg/m^3^) as reported for dairy cattle farm workers, in contrast to endotoxin concentrations, which were twice as high for goat farmers (360 EU/m^3^ in dairy cattle farm workers) (Basinas et al. 2014). We found the average inhalable dust exposure to be 3-fold lower for our goat farmers compared to previous research on pig farm workers (3.4 to 4.1 mg/m^3^), and for endotoxin exposure, this was almost 4-fold lower (2,100 to 2,400 EU/m^3^ in pig farm workers) (Bønløkke et al. 2012; Basinas et al. 2013). Personal exposure research for poultry farmers by Spaan et al. (Spaan et al. 2006) showed average inhalable dust concentrations to be almost 4-fold higher for broiler farmers (4.2 mg/m^3^) and 9-fold higher for laying hen farmers (9.5 mg/m^3^). Endotoxin exposure appeared to be similar for broiler farmers (880 EU/m^3^) and 3-fold higher for laying hen farmers (2,090 EU/m^3^) compared to our study with goat farmers. Results from studies on overall indoor PM_10_ pollution as measured by stationary air sampling demonstrate a similar pattern: considerable lower PM_10_ concentrations measured (between 0.04 and 0.27 mg/m^3^) inside goat farms compared to pig farms (0.42 to 1.09 mg/m^3^) and poultry farms (1.28 to 3.36 mg/m^3^), but vastly higher compared to dairy cattle farms (0.04 mg/m^3^) (Aarnink et al. 2011a, 2014; Winkel et al. 2015). Arguably, the distinctive husbandry system of intensive goat farming contributes to these findings. This is in line with previous studies indicating a large influence of specific farm characteristics which differed within and across livestock sectors (eg pig, poultry, and cattle) (Eduard et al. 2004; Samadi et al. 2009; Bønløkke et al. 2012; Basinas et al. 2013, 2014; Pfister et al. 2018). In contrast to dairy cows, goats are kept on deep-litter bedding, which represent a major source of PM and endotoxins. This deep-litter is not removed frequently (only once or twice per year), but instead a fresh layer of straw is added daily on top leading to accumulating bedding material with urine and feces. This facilitates microbial persistence and proliferation inside the goat farm, leading to relatively high PM and endotoxin exposures for goat farmers. The even higher occupational exposures observed among pig and poultry farmers can be attributed to the high intensity of animal production in enclosed housing systems with mechanical ventilation. Such systems can lead to the accumulation of indoor air pollution, particularly when ventilation is restricted during colder episodes to conserve energy. In contrast, natural ventilation systems in ruminant farming ensure a higher and more continuous air exchange (Cambra-López et al. 2011b).

In accordance with our observations, emission studies on goat farms also showed higher concentrations in mechanically ventilated houses compared to those with natural ventilation (Van der Giessen et al. 2024). Sampling of different PM size fractions on goat farms suggested lower endotoxin content in the PM_10_ fraction compared to larger size fractions (Winkel et al. 2014; Aarnink et al. 2014), whereas our personal exposure measurements indicated the opposite.

### Health implications

Our measurements showed that personal exposure to endotoxins exceeded the advised occupational exposure limit for most of the workdays (90%). Similar exceedances have also been reported among other livestock farmers (Liebers et al. 2006; Spaan et al. 2006; Farokhi et al. 2018). It is well known that exposure to endotoxins can lead to acute and chronic respiratory symptoms, chronic bronchitis, and accelerated decline in lung function (Liebers et al. 2008; Sigsgaard et al. 2020; Seidel et al. 2023). In our study, the highest endotoxin content was found in the PM_10_ fraction, which can reach the lower airways and therefore may contribute to more severe health effects (Garcia et al. 2013). Inhalable dust concentrations were generally below the previously applied Danish occupational exposure limit of 3 mg/m^3^ for the organic fraction of the total dust measurement (exceeded in only 1 out of 73 measurements) (Danish Working Environment Authority 2007). This observation, where exceedances of recommended limits are common for endotoxin but rare for inhalable dust, demonstrates that exposure assessment solely based on particulate matter is insufficient for accurately characterizing health risks in this occupational setting.

To protect goat farmers’ health, it is important to minimize exposure to PM and endotoxins as much as possible. Ultimately, source-level interventions are preferred, as better indoor air quality will also benefit animal health. Deep-litter housing likely contributes substantially to dust and microbial exposure, yet the impact of this and alternative husbandry systems requires further investigation. Meanwhile, measures that can be considered on the short term include proper use of personal respiratory protective equipment (eg respiratory protective masks, covering mouth and nose). This is especially advised when goat farm workers are performing typical dust generating tasks (Von Essen et al. 2010) such as straw handling, moving animals and cleaning out the deep litter boxes.

### Limitations

An important limitation of this study is the modest sample size. Recruitment required substantial effort, as many farmers were hesitant to participate due to ongoing public debates and societal concerns regarding the impacts of livestock farming on human health and the environment. Ultimately, the participating farms are representative of the typical range of herd size on Dutch goat farms, and the sampled working days reflected routine work activities.

Insights into temporal variation were limited as every farm was only visited twice, and no measurements were performed in January–May. So, despite these limitations, the study provides meaningful insights into the variation of exposure levels during typical work shifts on representative dairy goat farms. The sample size did not allow further inferential analyses of potentially relevant farm-level characteristics, like animal numbers, air flow, and indoor climate parameters, to explain between-farm variation.

An important potential limitation in field studies on microbial exposures is the risk of contamination and bacterial proliferation, necessitating proper procedures and controls. Previous comparisons in our laboratory of endotoxin recovery from filters stored at 4 degrees or 20 degrees prior to extraction showed that storage temperature had only a minimal effect (Spaan et al. 2007). Furthermore, all our field blanks were below the limit of detection for endotoxin.

### Future research

This study showed clear variation in occupational exposure levels between farms and workers. Therefore, future research should further elucidate which farm characteristics and work practices lead to lower personal exposures. A larger study including personal air sampling should be incorporated to accurately assess exposure related to specific tasks and routines.

Additionally, stationary air measurements are recommended as these allow for larger equipment enabling longer-term multiple size-fraction sampling. We focused on PM and endotoxins as risk factors for noninfectious health effects. However, in the goat farming industry, (zoonotic) infectious disease risks also deserve attention (Lokhorst et al. 2024; Cornu Hewitt et al. 2025; Van der Giessen et al. 2024). Well-known infectious diseases among goat farmers, such as Q fever, likely represent only a fraction of the overall occupational infectious disease risk. Moreover, increasing evidence from the Livestock Farming and Neighboring Residents’ Health study shows that respiratory infections occur more frequently in neighboring residents of goat farms (Smit et al. 2012; Freidl et al. 2017; Kalkowska et al. 2018; Post et al. 2019; Lotterman et al. 2023). More than 30 bacteria known to cause pneumonia were commonly detected at goat farms, a finding that warrant follow-up research (Van der Giessen et al. 2024). To better understand occupational and environmental health risks, there is an urgent need for deeper investigations at goat farms for improved (microbiological) characterization and quantification of aerosols combined with better understanding of their origination, dynamics and emissions. Given the anticipated global expansion of the goat farming industry amidst broader challenges for responsible production under climate change, future research should inform sustainable farm management and husbandry practices.

## Conclusions

This work showed that goat farm workers experience elevated exposures to particulate matter and endotoxins. This adds to previous research reporting high occupational exposures for intensive pig, poultry, and cattle farm workers. As almost all endotoxin levels measured exceeded the advised occupational exposure limit, adverse health effects are likely. There was a clear variation in exposure levels between farms and work profiles.

Further research on more farms and more detailed microbiological characterization of air samples is warranted to better explain these differences, as it will give directions toward husbandry and working practices associated with lower exposures. The number of intensively farmed goats is increasing and to safeguard health of workers, animals, and surrounding communities, this expansion should be accompanied by sustainable farming practices.

## Supplementary Material

wxag020_Supplementary_Data

## Data Availability

Data are not publicly available due to the privacy protection of participants. This is in agreement with the Medical Ethical Committee of the University Medical Centre of Utrecht, which approved the study protocol (number 13/533). Data can be made (partially) available upon reasonable request addressed to the corresponding author of this paper.

## References

[wxag020-B1] Aarnink AJA, Cambra-López M, Lai HTL, Ogink NWM. 2011b. Deeltjesgrootteverdeling en bronnen van stof in stallen. Report No.: ISSN 1570-8616, Rapport 452.

[wxag020-B2] Aarnink AJA et al 2011a. Emissies van stof en ziektekiemen uit melkgeitenstallen. Report No.: ISSN 1570-8616, Rapport 489.

[wxag020-B3] Aarnink AJA et al 2014. Emissies van stof en ziektekiemen uit melkgeitenstallen; aanvullende metingen. Report No.: ISSN 1570-8616, Rapport 712.

[wxag020-B4] American Thoracic Society . 2012. Respiratory Health Hazards in Agriculture. American Journal of Respiratory and Critical Care Medicine. [published online ahead of print] [accessed 2024 Aug 20]. https://www.atsjournals.org/doi/10.1164/ajrccm.158.supplement_1.rccm1585s1

[wxag020-B5] Basinas I et al 2013. Exposure to inhalable dust and endotoxin among Danish pig farmers affected by work tasks and stable characteristics. Ann Occup Hyg. 57:1005–1019. 10.1093/annhyg/met029. 23792973

[wxag020-B6] Basinas I et al 2014. Exposure-affecting factors of dairy farmers’ exposure to inhalable dust and endotoxin. Ann Occup Hyg. 58:707–723. 10.1093/annhyg/meu024. 24748620

[wxag020-B7] Basinas I et al 2015. A comprehensive review of levels and determinants of personal exposure to dust and endotoxin in livestock farming. J Expo Sci Environ Epidemiol. 25:123–137. 10.1038/jes.2013.83. 24280684

[wxag020-B8] Bønløkke JH et al 2012. Work-related health effects in swine building workers after respiratory protection use. J Occup Environ Med. 54:1126–1132. 10.1097/JOM.0b013e31825461f4. 22918380

[wxag020-B9] Cambra-López M et al 2010. Airborne particulate matter from livestock production systems: a review of an air pollution problem. Environ Pollut. 158:1–17. 10.1016/j.envpol.2009.07.011. 19656601

[wxag020-B10] Cambra-López M, Hermosilla T, Lai HTL, Aarnink AJA, Ogink NWM. 2011a. Particulate matter emitted from poultry and pig houses: source identification and quantification. Transac ASABE/Am Soc Agricult Biol Eng. 54:629–642. 10.13031/2013.36466.

[wxag020-B11] Cambra-López M, Torres AG, Aarnink AJA, Ogink NWM. 2011b. Source analysis of fine and coarse particulate matter from livestock houses. Atmos Environ. 45:694–707. 10.1016/j.atmosenv.2010.10.018.

[wxag020-B12] Cornu Hewitt B et al 2025. Occupational and environmental livestock exposures are associated with alterations in the upper respiratory tract microbiome. Environ Res. 286:122847. 10.1016/j.envres.2025.122847. 40946892

[wxag020-B13] Danish Working Environment Authority . 2007. Arbejdstilsynet [Danish Working Environment Authority] At-vejledning. (2007) Grænseværdier for stoffer og materialer. [Limit values for substances and materials], Publication no. C.0.1. The Danish Working Environment Authority. [accessed 2025 Sept 26]. https://at.dk/regler/atvejledninger/graensevaerdier-stoffer-materialer-c-0-1/

[wxag020-B14] Dungan RS . 2010. BOARD-INVITED REVIEW: fate and transport of bioaerosols associated with livestock operations and manures. J Anim Sci. 88:3693–3706. 10.2527/jas.2010-3094. 20622180 PMC7109640

[wxag020-B15] Eduard W, Douwes J, Omenaas E, Heederik D. 2004. Do farming exposures cause or prevent asthma? Results from a study of adult Norwegian farmers. Thorax. 59:381–386. 10.1136/thx.2004.013326. 15115863 PMC1747014

[wxag020-B16] Eduard W, Pearce N, Douwes J. 2009. Chronic bronchitis, COPD, and lung function in farmers: the role of biological agents. Chest. 136:716–725. 10.1378/chest.08-2192. 19318669

[wxag020-B17] FAO , 2017. Global Livestock Environmental Assessment Model. FAO Publ. Acc (Gleam). [accessed 2023 May 01] https://Www.Fao.Org/Gleam/En/.

[wxag020-B18] FAOSTAT . [accessed 2024 Aug 23]. https://www.fao.org/faostat/en/#rankings/countries_by_commodity

[wxag020-B19] Farokhi A, Heederik D, Smit LAM. 2018. Respiratory health effects of exposure to low levels of airborne endotoxin—a systematic review. Environ Health. 17:14. 10.1186/s12940-018-0360-7. 29422043 PMC5806377

[wxag020-B20] Freidl GS et al 2017. Livestock-associated risk factors for pneumonia in an area of intensive animal farming in The Netherlands. PLoS One. 12:e0174796. 10.1371/journal.pone.0174796. 28362816 PMC5376295

[wxag020-B21] Garcia J et al 2013. Occupational exposure to particulate matter and endotoxin for California dairy workers. Int J Hyg Environ Health. 216:56–62. 10.1016/j.ijheh.2012.04.001. 22579491

[wxag020-B22] Guillien A, Soumagne T, Dalphin J-C, Degano B. 2019. COPD, airflow limitation and chronic bronchitis in farmers: a systematic review and meta-analysis. Occup Environ Med. 76:58–68. 10.1136/oemed-2018-105310. 30482880

[wxag020-B23] Health Council of the Netherlands: Dutch Expert Committee on Occupational Standards (DECOS) . 1998. Endotoxins. Health Council of the Netherlands. publication no. 1998/03WGD.

[wxag020-B24] IRAS . Weighing of teflon filters to determine particle mass concentrations. Escape Project Manual. 2009 [accessed 2025 February 4]. http://Www.Escapeproject.Eu/Manuals/ESCAPE-Weighing-Sop.Pdf. 2009.

[wxag020-B25] Ismoyowati SJ . 2019. Duck production for food security. IOP Conf Ser Earth Environ Sci. 372:012070. 10.1088/1755-1315/372/1/012070.

[wxag020-B26] Kalkowska DA et al 2018. Associations between pneumonia and residential distance to livestock farms over a five-year period in a large population-based study. PLoS One. 13:e0200813. 10.1371/journal.pone.0200813. 30016348 PMC6049940

[wxag020-B27] Kirychuk et al 2010. Endotoxin and Dust at Respirable and Nonrespirable Particle Sizes are not Consistent Between Cage- and Floor-Housed Poultry Operations. The Annals of Occupational Hygiene. [published online ahead of print] [accessed 2026 Jan 27]. https://academic.oup.com/annweh/article/54/7/824/202799/Endotoxin-and-Dust-at-Respirable-and-Nonrespirable 10.1093/annhyg/meq047 20538718

[wxag020-B28] Kuznetsova A, Brockhoff PB, Christensen RHB. 2017. **lmerTest** package: tests in linear mixed effects models. J Stat Soft. 82:1–26. 10.18637/jss.v082.i13.

[wxag020-B29] Liebers V, Brüning T, Raulf-Heimsoth M. 2006. Occupational endotoxin-exposure and possible health effects on humans (review). Am J Ind Med. 49:474–491. 10.1002/ajim.20310. 16586405

[wxag020-B30] Liebers V, Brüning T, Raulf M. 2020. Occupational endotoxin exposure and health effects. Arch Toxicol. 94:3629–3644. 10.1007/s00204-020-02905-0. 32910236

[wxag020-B31] Liebers V, Raulf-Heimsoth M, Brüning T. 2008. Health effects due to endotoxin inhalation (review). Arch Toxicol. 82:203–210. 10.1007/s00204-008-0290-1. 18322674

[wxag020-B32] Lokhorst W et al 2024. Literature review on micro-organisms from domestic goats potentially causing human pneumonia. Infect Ecol Epidemiol. 14:2406835. 10.1080/20008686.2024.2406835. 39539745 PMC11559867

[wxag020-B33] Lotterman A et al 2023. Increased risk of pneumonia amongst residents living near goat farms in different livestock-dense regions in The Netherlands. PLoS One. 18:e0286972. 10.1371/journal.pone.0286972. 37405987 PMC10321607

[wxag020-B34] Nazan Koluman D . 2023. Goats and their role in climate change. Small Rumin Res. 228:107094. 10.1016/j.smallrumres.2023.107094.

[wxag020-B35] Pfister H et al 2018. Factors determining the exposure of dairy farmers to thoracic organic dust. Environ Res. 165:286–293. 10.1016/j.envres.2018.04.031. 29758401

[wxag020-B36] Poole JA, Romberger DJ. 2012. Immunological and inflammatory responses to organic dust in agriculture. Curr Opin Allergy Clin Immunol. 12:126–132. 10.1097/ACI.0b013e3283511d0e. 22306554 PMC3292674

[wxag020-B37] Portengen L et al 2005. Endotoxin exposure and atopic sensitization in adult pig farmers. J Allergy Clin Immunol. 115:797–802. 10.1016/j.jaci.2004.11.046. 15806001

[wxag020-B38] Posit team . 2025. Rstudio. [accessed 2025 Aug 8]. http://www.posit.co/

[wxag020-B39] Post PM et al 2019. Risk of pneumonia among residents living near goat and poultry farms during 2014-2016. PLoS One. 14:e0223601. 10.1371/journal.pone.0223601. 31609989 PMC6791541

[wxag020-B40] Pragna P et al 2018. Climate change and goat production: enteric methane emission and its mitigation. Animals (Basel). 8:235. 10.3390/ani8120235. 30544616 PMC6316019

[wxag020-B41] Samadi S et al 2009. Exposure to inhalable dust, endotoxins, β(1→3)-glucans, and airborne microorganisms in horse stables. Ann Occup Hyg. 53:595–603. 10.1093/annhyg/mep040. 19561032

[wxag020-B42] Seidel J et al 2023. Lessons from dairy farmers for occupational allergy and respiratory disease. Curr Allergy Asthma Rep. 23:325–339. 10.1007/s11882-023-01081-2. 37191901 PMC10186320

[wxag020-B43] Sigsgaard T et al 2020. Respiratory diseases and allergy in farmers working with livestock: a EAACI position paper. Clin Transl Allergy. 10:29. 10.1186/s13601-020-00334-x. 32642058 PMC7336421

[wxag020-B44] Smit LAM et al 2012. Q fever and pneumonia in an area with a high livestock density: a large population-based study. PLoS One. 7:e38843. 10.1371/journal.pone.0038843. 22685612 PMC3369851

[wxag020-B45] Spaan S et al 2006. Exposure to inhalable dust and endotoxins in agricultural industries. J Environ Monit. 8:63–72. 10.1039/B509838F. 16395461

[wxag020-B46] Spaan S et al 2008. Effect of extraction and assay Media on analysis of airborne endotoxin. Appl Environ Microbiol. 74:3804–3811. 10.1128/AEM.02537-07. 18441112 PMC2446558

[wxag020-B47] Spaan S, Heederik DJJ, Thorne PS, Wouters IM. 2007. Optimization of airborne endotoxin exposure assessment: effects of filter type, transport conditions, extraction solutions, and storage of samples and extracts. Appl Environ Microbiol. 73:6134–6143. 10.1128/AEM.00851-07. 17675430 PMC2075030

[wxag020-B48] Van der Giessen J et al 2024. Veehouderij en gezondheid omwonenden (VGO-III). Actualisatie epidemiologische studies 2014-2019. Onderzoek naar longontstekingen rond geitenhouderijen 2018–2024. RIVM-rapport 2024-0167.

[wxag020-B49] Von Essen S, Moore G, Gibbs S, Larson KL. 2010. Respiratory issues in beef and pork production: recommendations from an expert panel. J Agromedicine. 15:216–225. 10.1080/1059924X.2010.486283. 20665307

[wxag020-B50] Wallace L . 2000. Correlations of personal exposure to particles with outdoor air measurements: a review of recent studies. Aerosol Sci Technol. 32:15–25. 10.1080/027868200303894.

[wxag020-B51] Winkel A et al 2015. Emissions of particulate matter from animal houses in The Netherlands. Atmos Environ. 111:202–212. 10.1016/j.atmosenv.2015.03.047.

[wxag020-B52] Winkel A, Wouters IM, Aarnink AJA, Heederik DJJ, Ogink NWM. 2014. Emissies van endotoxinen uit de veehouderij: een literatuurstudie voor ontwikkeling van een toetsingskader. Rapport 773.

[wxag020-B53] WSER , 2025. Holdings, animals and farm size—Goat farming (web page). www.agrimatie.nl. Wageningen Social & Economic Research (WSER), Wageningen, the Netherlands. 2025. [accessed 2025 Feb 13]. https://agrimatie.nl/ThemaResultaat.aspx?subpubID=2232&themaID=2286&sectorID=2238&indicatorID=2015

