# Supplementary material for: Occupational exposure of goat farm workers to particulate matter and endotoxin
Source: Ann Work Expo Health. 2026 Apr 6;70(3):wxag020. doi: 10.1093/annweh/wxag020 (PMC13056540; doi:10.1093/annweh/wxag020)
Supplement: wxag020_Supplementary_Data [file wxag020_supplementary_data.pdf]

Supplements to paper

Title

Occupational exposure of goat farm workers to particulate matter and endotoxin

Authors

Aniek Lotterman[1], Ifeoluwa Olufotebi[1], Inge M. Wouters[1], Albert Winkel[2], Lidwien A.M. Smit[1], Myrna M.T. de Rooij[1]

Affiliations:

[1] Institute for Risk Assessment Sciences, Utrecht University, Utrecht, the Netherlands

[2] Wageningen Livestock Research, Wageningen, the Netherlands

Table S.1 Summary of tasks based on sources of exposure

Source of exposure	Tasks
**Inside stables**	
Feeding	Automatic feeding, feed mixer, manual feeding, mixing milk for lambs
Cleaning	Cleaning: newborn crates, lamb stall before weaning, lamb stall after weaning, milk installation, milk stall, milking waiting area, food related items and places
Pot handling	Emptying: lamb pot before weaning, lamb pot after weaning, goats pot stall
Straw handling	Automatic straw spreading, manual straw spreading
Direct animal contact	Care for lambs, help with birth, care for dairy goat, direct animal contact
Others	Pest control, indoor installation, and repair
**Outside stables**	
Indoor activities	Administrative work, breaks, working inside the house, working in indoor shed
Outdoor activities	Repair and maintenance, arable farming, general outside work, pest control, garden pasture field
Manure handling	Manure handling
Food preparation	feed extraction, mixing feed, silage preparation, replenishing feed mix,

All tasks were included in the task form that participants filled out for each sampling moment

Table S.2 proportion of time spent on tasks, per sample moment per participant, per farm

Farm	Participant	Visit	Inside stables	Feeding	Cleaning	Pot_handling	Straw_handling	Milking_goats	Direct_animal_contact	Others	Outside stables	Manure_processing	Indoor_activities	Outdoor_activities	Feed_preparation
01	01	1	0,60	0,14	0,04	0,00	0,03	0,22	0,05	0,00	0,40	0,00	0,26	0,00	0,06
		2	0,58	0,21	0,06	0,00	0,06	0,24	0,00	0,00	0,42	0,00	0,19	0,22	0,00
	02	1	0,50	0,15	0,06	0,00	0,03	0,24	0,00	0,00	0,50	0,00	0,24	0,24	0,00
		2	0,55	0,21	0,06	0,00	0,00	0,24	0,00	0,00	0,45	0,00	0,24	0,18	0,00
02	03	1	0,93	0,00	0,17	0,00	0,00	0,77	0,00	0,00	0,07	0,00	0,07	0,00	0,00
		2	0,90	0,00	0,19	0,00	0,00	0,71	0,00	0,00	0,10	0,00	0,10	0,00	0,00
	04	1	0,87	0,00	0,00	0,00	0,04	0,53	0,29	0,00	0,13	0,00	0,13	0,00	0,00
	05	1	0,84	0,52	0,02	0,00	0,00	0,14	0,00	0,00	0,16	0,00	0,13	0,00	0,00
		2	0,65	0,45	0,05	0,00	0,05	0,09	0,02	0,00	0,35	0,00	0,35	0,00	0,00
	10	2	0,30	0,00	0,00	0,00	0,00	0,03	0,07	0,00	0,70	0,00	0,00	0,24	0,00
03	06	1	0,74	0,09	0,25	0,00	0,06	0,30	0,00	0,00	0,26	0,00	0,15	0,00	0,09
		2	0,60	0,20	0,00	0,00	0,00	0,40	0,00	0,00	0,40	0,00	0,16	0,00	0,24
	07	1	0,67	0,33	0,17	0,00	0,17	0,00	0,00	0,00	0,33	0,00	0,33	0,00	0,00
		2	0,44	0,21	0,00	0,00	0,12	0,00	0,00	0,00	0,56	0,00	0,17	0,00	0,25
04	08	1	0,72	0,00	0,03	0,00	0,05	0,44	0,20	0,00	0,28	0,04	0,24	0,00	0,00
		2	0,56	0,04	0,02	0,00	0,00	0,40	0,05	0,00	0,44	0,00	0,40	0,00	0,00
	09	1	0,33	0,00	0,00	0,00	0,17	0,00	0,13	0,00	0,67	0,00	0,48	0,00	0,13
		2	0,54	0,00	0,00	0,00	0,08	0,00	0,46	0,00	0,46	0,00	0,25	0,00	0,21
05	11	1	0,86	0,00	0,14	0,00	0,00	0,71	0,00	0,00	0,14	0,00	0,14	0,00	0,00
		2	1,00	0,00	0,18	0,00	0,00	0,81	0,00	0,00	0,00	0,00	0,00	0,00	0,00
	12	1	0,80	0,24	0,00	0,00	0,04	0,12	0,30	0,00	0,20	0,00	0,18	0,00	0,00
		2	1,00	0,52	0,00	0,00	0,10	0,19	0,19	0,00	0,00	0,00	0,00	0,00	0,00
06	13	1	0,96	0,00	0,05	0,00	0,00	1,00	0,00	0,00	0,04	0,00	0,04	0,00	0,00
		2	0,96	0,00	0,05	0,00	0,00	0,97	0,00	0,00	0,04	0,00	0,04	0,00	0,00
	14	1	0,86	0,38	0,00	0,00	0,33	0,07	0,15	0,00	0,14	0,07	0,07	0,00	0,00
	22	2	1,00	0,20	0,12	0,00	0,19	0,03	0,15	0,00	0,00	0,00	0,00	0,00	0,00
07	15	1	0,58	0,11	0,32	0,00	0,11	0,00	0,00	0,00	0,42	0,00	0,11	0,00	0,29
		2	0,20	0,00	0,00	0,00	0,07	0,00	0,13	0,00	0,80	0,00	0,27	0,33	0,20
	17	1	0,94	0,00	0,45	0,00	0,23	0,00	0,23	0,00	0,06	0,00	0,06	0,00	0,00
		2	0,29	0,00	0,00	0,00	0,14	0,00	0,14	0,00	0,71	0,12	0,28	0,28	0,00
	18	1	1,00	0,00	0,04	0,00	0,00	1,00	0,00	0,00	0,00	0,00	0,00	0,00	0,00
		2	1,00	0,00	0,04	0,00	0,00	0,67	0,00	0,00	0,00	0,00	0,00	0,00	0,00
	19	1	0,50	0,00	0,21	0,00	0,14	0,00	0,14	0,00	0,50	0,00	0,14	0,35	0,00
		2	0,41	0,00	0,00	0,00	0,14	0,00	0,14	0,00	0,59	0,12	0,14	0,14	0,00
	20	2	0,11	0,00	0,00	0,00	0,09	0,00	0,00	0,00	0,89	0,00	0,36	0,40	0,00
	21	2	0,08	0,00	0,00	0,00	0,04	0,00	0,04	0,00	0,92	0,08	0,32	0,53	0,00

08	23	1	1,00	0,00	0,12	0,36	0,15	0,13	0,00	0,15	0,00	0,00	0,00	0,00	0,00
		2	0,71	0,00	0,07	0,00	0,13	0,20	0,00	0,17	0,29	0,00	0,24	0,00	0,00
	24	1	0,87	0,00	0,12	0,00	0,00	0,37	0,00	0,04	0,13	0,00	0,08	0,00	0,00
		2	0,84	0,00	0,09	0,00	0,02	0,35	0,00	0,24	0,16	0,00	0,14	0,00	0,00
	25	1	1,00	0,23	0,07	0,00	0,00	0,37	0,00	0,33	0,00	0,00	0,00	0,00	0,00
		2	1,00	0,40	0,06	0,00	0,00	0,40	0,00	0,17	0,00	0,00	0,00	0,00	0,00
09	28	1	0,67	0,32	0,00	0,00	0,00	0,00	0,00	0,00	0,33	0,16	0,00	0,00	0,00
	30	1	1,00	0,00	0,00	0,00	0,00	0,98	0,00	0,00	0,00	0,00	0,00	0,00	0,00
		2	1,00	0,00	0,12	0,00	0,00	0,88	0,00	0,00	0,00	0,00	0,00	0,00	0,00
	31	1	0,44	0,07	0,07	0,00	0,00	0,14	0,02	0,00	0,56	0,00	0,35	0,03	0,00
		2	0,87	0,16	0,05	0,00	0,05	0,44	0,00	0,00	0,13	0,00	0,11	0,00	0,00
10	33	1	0,64	0,08	0,00	0,06	0,00	0,37	0,00	0,00	0,36	0,00	0,21	0,08	0,00
		2	0,71	0,00	0,16	0,08	0,00	0,37	0,00	0,00	0,29	0,00	0,00	0,25	0,00
	34	1	0,43	0,47	0,00	0,00	0,00	0,00	0,00	0,00	0,57	0,00	0,42	0,21	0,00
		2	0,67	0,65	0,00	0,00	0,00	0,00	0,00	0,00	0,33	0,00	0,00	0,33	0,00
11	35	1	0,49	0,04	0,00	0,19	0,01	0,39	0,05	0,00	0,51	0,00	0,27	0,31	0,12
		2	0,72	0,15	0,00	0,00	0,05	0,10	0,14	0,00	0,28	0,00	0,13	0,00	0,04
	36	1	0,55	0,21	0,00	0,00	0,04	0,00	0,06	0,12	0,45	0,00	0,27	0,04	0,04
		2	0,60	0,09	0,00	0,00	0,00	0,39	0,09	0,00	0,40	0,00	0,26	0,00	0,12
12	37	1	0,84	0,14	0,03	0,00	0,03	0,35	0,20	0,00	0,16	0,00	0,15	0,00	0,00
		2	0,85	0,47	0,00	0,00	0,18	0,00	0,00	0,00	0,15	0,00	0,12	0,00	0,00
	38	1	0,82	0,29	0,00	0,00	0,12	0,00	0,26	0,00	0,18	0,00	0,15	0,00	0,00
		2	0,82	0,28	0,10	0,00	0,00	0,42	0,00	0,00	0,18	0,00	0,12	0,00	0,06
13	39	1	0,53	0,09	0,00	0,00	0,00	0,43	0,00	0,00	0,47	0,00	0,12	0,34	0,00
		2	0,47	0,00	0,00	0,00	0,00	0,50	0,00	0,00	0,53	0,00	0,14	0,32	0,11
	40	1	0,59	0,02	0,09	0,00	0,00	0,46	0,00	0,00	0,41	0,00	0,06	0,34	0,00
		2	0,52	0,00	0,00	0,00	0,00	0,48	0,00	0,00	0,48	0,00	0,07	0,36	0,00
14	41	1	1,00	0,67	0,00	0,00	0,17	0,00	0,17	0,00	0,00	0,00	0,00	0,00	0,00
		2	0,74	0,32	0,00	0,00	0,00	0,00	0,17	0,00	0,26	0,00	0,17	0,00	0,00
	42	1	1,00	0,00	0,15	0,00	0,00	0,85	0,00	0,00	0,00	0,00	0,00	0,00	0,00
		2	1,00	0,00	0,11	0,00	0,00	0,89	0,00	0,00	0,00	0,00	0,00	0,00	0,00
15	43	1	0,85	0,00	0,12	0,33	0,00	0,41	0,00	0,00	0,15	0,00	0,15	0,00	0,00
	44	1	0,70	0,08	0,06	0,29	0,05	0,21	0,00	0,00	0,30	0,00	0,18	0,12	0,00
		2	0,83	0,09	0,00	0,00	0,00	0,32	0,00	0,21	0,17	0,00	0,13	0,00	0,00
	45	2	0,75	0,00	0,12	0,00	0,00	0,14	0,00	0,45	0,25	0,00	0,18	0,05	0,00

Table S.3 Characteristics per goat farm

Farm Characteristics	Goat Farms														
	01	02	03	04	05	06	07	08	09	10	11	12	13	14	15
**Type of farm**															
Milking	x	x	x	x	x	x	x	x	x	x	x	x	x	x	x
Breeding	x	x		x	x		x	x				x			
Meat			x	x	x	x	x	x		x		x			
Organic farming			x												
Continued milking	x	x	x	x	x	x	x	x	x		x	x	x	x	x
No. houses	3	2	3	2	3	2	5	5	2	3	4	3	5	2	3
Herd size	960	1960	110[a]	1400	1840	2300	2800	1943	1850	368	1304	>1700	1100	850	1200
**Feed**															
Grass		x					x	x							
Grass silage			x	x	x	x			x	x	x	x	x	x	x
Concentrate	x	x	x	x					x	x	x	x	x		
Mais	x	x			x			x							
Hay									x			x		x	
(Rapeseed) straw						x		x		x				x	x
Alfalfa	x		x			x	x	x	x						
Pulp		x		x	x	x	x	x	x			x			
**Method of feeding**															
Manually			x							x			x		x
Feed Wagon	x	x			x		x				x	x	x	x	
Feed robot				x				x	x	x					x
**Manure storage**															
- **Method**															
Covered (air permeable or airtight)	x	x	x	x	x	x						x			x
Roofed							x								
Uncovered/ open					x				x	x	x			x	
In stable/ silo	x	x						x					x		
- **Duration**															
≤ 2 months		x		x				x	x			x		x	
2-4 months	x				x	x							x		x
4-6 months							x			x	x				
> 6 months			x						x						
**Ventilation system**															
All Mechanical						x		x	x						
Mixed	x	x	x	x	x		x				x	x	x		
All Natural										x				x	x

[a] number not provided on the form for farm characteristics, but retrieved from the 2018 survey data

Table S.4 Associations between work profiles with each of the four exposure outcomes assessed in a mixed model

	Compared to 'main milking'	Est. (CI*)	p-value*
**Inhalable dust fraction**			
**PM mass**	'Also milking'	-361 (-813 – 90)	0.125
	'No milking'	-303 (-815 – 210)	0.255
**Endotoxin**	'Also milking'	-908 (-1344 – -471)	**< 0.001**
	'No milking'	-368 (-856 – 119)	0.148
**PM_{10} fraction**			
**PM mass**	'Also milking'	-244 (-413 – -74)	**0.008**
	'No milking'	-81 (-278 – 116)	0.424
**Endotoxin**	'Also milking'	-647 (-1036 – -258)	**0.002**
	'No milking'	-454 (-902 – -5.6)	*0.052*

Mixed model for all four exposure outcomes with work profile as fixed effect ('main milking' as reference/ indicator) and random intercepts for farm and participant.

All estimates are negative because, compared to the work profile main milking, there are lower exposure levels for 'also milking' and 'no milking' work profiles.

* 95% CIs (Confidence Intervals) and p-values were based on Satterthwaite's approximation

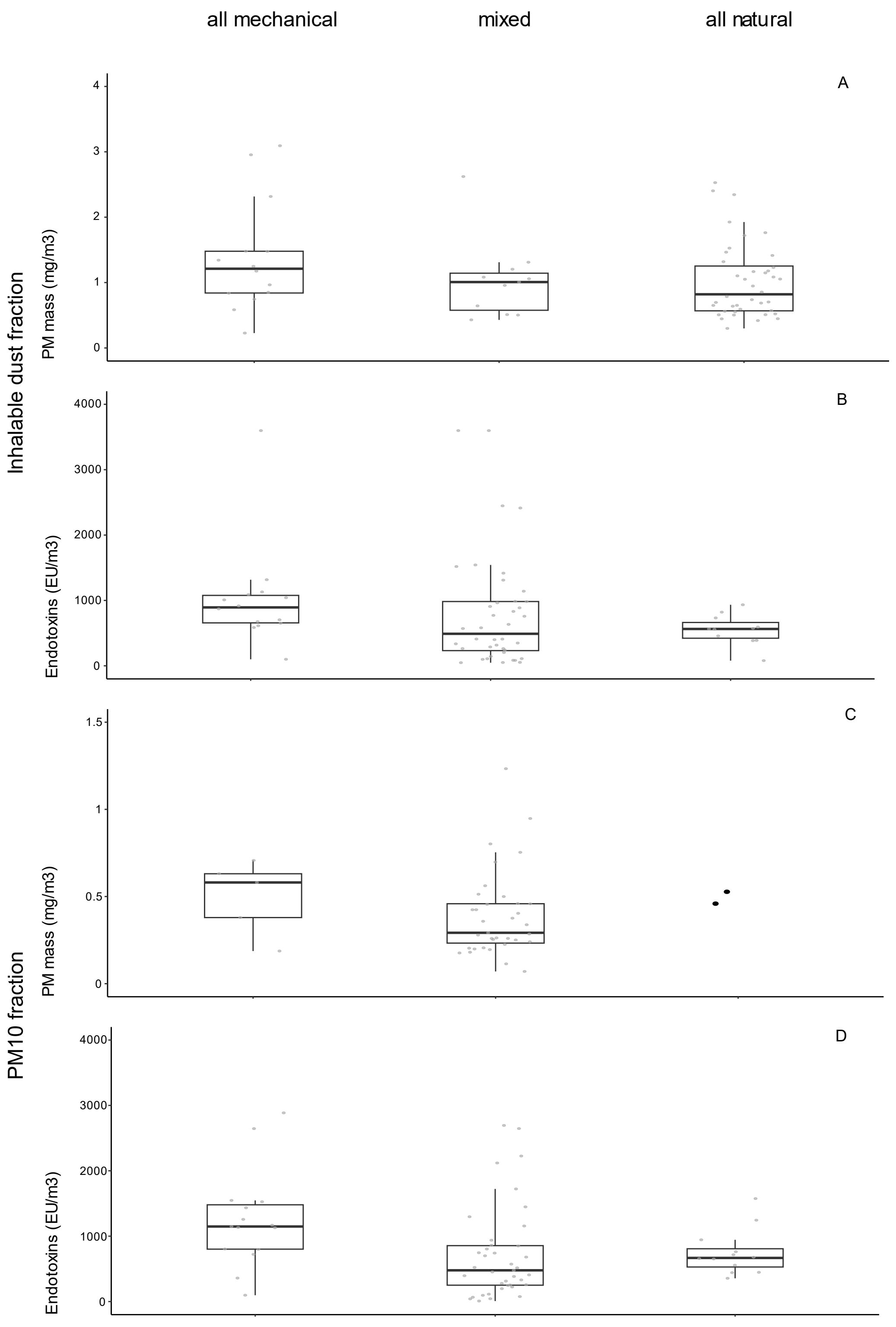

Figure S.1 Box plots for each of the exposure outcomes per ventilation category

For the inhalable dust fraction PM mass is shown in panel A and endotoxins in panel B, and for the PM_{10} fraction PM mass is shown in panel C and endotoxins in panel D.

PM_{10} PM mass has only 2 successful measurements in the 'all natural' category and is therefore not depicted in a box plot.
Testing for differences per exposure outcome between ventilation type showed bordeline significant differences (p = 0.056) for endotoxin in the PM_{10} fraction between mixed vs all mechanical ventilation (est. -618).
